# Multidrug-related protein 1 (MRP1) polymorphisms rs129081, rs212090, and rs212091 predict survival in normal karyotype acute myeloid leukemia

**DOI:** 10.1007/s00277-020-04163-7

**Published:** 2020-07-03

**Authors:** Desiree Kunadt, Christian Dransfeld, Claudia Dill, Maria Schmiedgen, Michael Kramer, Heidi Altmann, Christoph Röllig, Martin Bornhäuser, Ulrich Mahlknecht, Markus Schaich, Friedrich Stölzel

**Affiliations:** 1 Department of Internal Medicine I, University Hospital Carl Gustav Carus, Technical University of Dresden, Fetscherstraße 74, 01307 Dresden, Germany; 2 Department of Internal Medicine, Division of Immunotherapy and Gene Therapy, José Carreras Research Centre, Saarland University Medical Centre, Homburg, Saar Germany; 3 Department of Internal Medicine Hematology/Oncology, St. Lukas Klinik, Solingen, Germany; 4 Department of Hematology, Oncology and Palliative Care, Rems-Murr-Klinikum, Winnenden, Germany

**Keywords:** Acute myeloid leukemia, Multidrug resistance, MRP1, SNPs, Prognosis, Survival

## Abstract

Resistant disease is still a main obstacle in acute myeloid leukemia (AML) treatment. Therefore, individual genetic variations affecting therapy response are gaining increasing importance. Both SNPs and ABC transporter genes could already be associated with drug resistance. Here, we report allelic variants of *MRP1* (*ABCC1*) SNPs rs129081, rs212090, and rs212091 with significant influences on survival in AML patients. DNA was extracted from bone marrow samples (*n* = 160) at diagnosis. Genotyping 48 SNPs within seven different ABC transporter genes using real-time PCR revealed rs129081 GG variant with a significant higher OS (*p* = 0.035) and DFS (*p* = 0.01). Comparing TT and AA rs212090 variants showed significant influences on DFS (*p* = 0.021). SNP rs212091 GG expression was associated with worse OS (*p* = 0.006) and a significant difference in DFS between alleles GG and AA (*p* = 0.018). The multivariable models confirmed a significant influence on OS for rs212091 (AA HR = 0.296, 95% CI 0.113–0.774, *p* = 0.013 and GG *p* = 0.044). Rs129081 variant CG, TT of rs212090, AA, and AG of rs212091 demonstrated significant impact on DFS (*p* = 0.024, *p* = 0.029, *p* = 0.017, and *p* = 0.042, respectively). This analysis demonstrates a significant influence of *MRP1* SNPs on survival in AML. As they were not associated to prognostic characteristics, we suggest these SNPs to be independent prognostic markers for AML.

**Electronic supplementary material:**

The online version of this article (10.1007/s00277-020-04163-7) contains supplementary material, which is available to authorized users.

## Background

Acute myeloid leukemia (AML) is characterized by uncontrolled proliferation of undifferentiated myeloid blasts requiring rapid induction chemotherapy to induce a remission. Although approximately 75% of patients achieve complete remission (CR) after induction treatment consisting of cytarabine (100 mg/m^2^, days 1–7) and daunorubicin (60 mg/m^2^, on three consecutive days), two-thirds of AML patients under the age of 60 relapse after successful induction chemotherapy, with an even higher rate in older patients [1–3]. Drug resistance affecting standard chemotherapeutic compounds in AML results in induction failure or relapse. Very often relapse presents with a highly aggressive AML clone insensitive to salvage chemotherapy with the urgent need of intensified treatment and more invasive procedures like allogeneic stem cell transplantation, thus far the only available curative option for these patients.

Molecular mechanisms causing therapy failure leading to inferior prognosis of AML patients are still incompletely understood and one of the most difficult obstacles in AML therapy. Within the context of clinically relevant inter-individual differences in treatment response or susceptibility to cytostatic agents and other drugs alike, single nucleotide polymorphisms (SNPs) were identified as individual genetic variations accountable for treatment failure. Several of these polymorphisms have already been described with different therapy outcomes, individual alterations of pharmaceutical responses like an elevated risk of chemoresistance, and risk to develop a malignancy in general and AML alike [4–8]. For example, SNPs located within cytarabine metabolizing enzyme genes were associated with therapeutic effects concerning AML patients’ outcome [9]. Illmer et al. could further demonstrate a significant impact of *ABCB1* (*MDR1*, *P-glycoprotein*) SNPs on therapy outcome in AML patients [4].

Transmembrane ATP-binding cassette transporters (ABC transporters) are important for substrate efflux and therefore providing a physiological function in multiple tissues, protecting cells against toxic metabolites. Some ABC transporters have certain substrate specificities for anticancer drugs, especially for cytarabine and daunorubicin. For cytarabine experimental analyses revealed that certain ABC transporter expressions lead to lower drug levels in leukemic blasts and thereby causing poor therapy response or even drug resistance [10].

ABC transporters have been extensively associated with the underlying principle of a so-called multidrug resistance (MDR), causing resistance to both multiple physiologic substrates and therapeutic drugs alike, leading to therapy failure, disease progression, and resistant disease [11–13].

ABC transporters ABCB1, ABCC1 (MRP1), and ABCG2 (BCRP) could be identified as the main MDR-generating transporters within the ABC family characterized by a very broad range of substrates and substrate specifity [11, 12, 14]. Furthermore, ABCC11 (MRP8) mRNA expression in blast progenitor cells of AML patients was demonstrated to have a significant influence on treatment response and long-term survival by conferring resistance to cytarabine [15].

Hence, the goal of this study was to further investigate whether so far non-investigated SNPs located within ABC-transporter genes responsible for daunorubicin efflux have an impact on treatment outcome in intensively treated younger AML patients.

## Patients and methods

This analysis included AML patients (non-APL AML) within the prospective AML2003 trial (NCT00180102) for patients under the age of 60 years with a normal karyotype (NK) from whom bone marrow aspirate with sufficient DNA extraction was available. Bone marrow aspirates obtained at diagnosis from a total of 160 Caucasian AML patients according to WHO criteria were investigated [16]. Written informed consent was obtained from all patients and the study was approved by the local ethics committee of the University of Dresden (EK153092003). The patients’ characteristics are summarized in Table 1. The median age at diagnosis was 45 years (IQR 38–54 years). Only patients with a normal karyotype were selected for this study, thereby minimizing heterogeneity of the underlying data set.

**Table 1:** Patients’ characteristics

Characteristics *n* = 160	*N* (%)	Median (range)
Sex		
Female	82 (51)	
Male	78 (49)	
Age at diagnosis (years)		46 (18–60)
White blood cell count (×10^9^/l)		31.2 (0.3–353)
Bone marrow blasts (%)		71 (8–95.5)
CD34 expression positive (%)		6 (0–93)
Lactat dehydrogenase (IU/l)		569 (167–5184)
Peroxidase expression positive		65 (1–100)
ECOG		
0/1	122 (76.3)	
2/3	29 (18.1)	
4/5	1 (0.6)	
Missing	8 (5)	
FAB subtype		
M0	2 (1.3)	
M1	50 (31.3)	
M2	46 (28.7)	
M4	25 (15.6)	
M5	22 (13.7)	
M6	2 (1.3)	
M7	1 (0.6)	
Missing	12 (7.5)	
Disease status		
De novo AML	149 (93.1)	
Therapy-related AML	3 (1.9)	
Secondary AML (preceeding MDS)	8 (5)	
*FLT3*-ITD mutation status		
Mutated	68 (42.5)	
Wildtype	92 (57.5)	
*NPM1* mutation status		
Mutated	89 (55.6)	
Wildtype	71 (44.4)	
CR	125 (78.1)	
No CR	22 (13.8)	
Missing	13 (8.1)	

*ECOG*, Eastern Co-operative Oncology Group performance index; *FAB*, French American British classification of acute leukemia; *AML*, acute myeloid leukemia; *MDS*, myelodysplastic syndrome; *FLT3*-ITD, *FMS-*like tyrosine kinase 3 internal tandem duplication; *NPM1*, Nucleophosmin 1; *CR*, complete remission

All patients received double induction chemotherapy with daunorubicin (60 mg/m^2^, on three consecutive days) and cytarabine (100 mg/m^2^, days 1–7). Complete remission (CR) was evaluated according to standard criteria [17]. A total of 78% of the patients achieved CR after standard induction treatment (Table 1). Adverse side effects and organ toxicity were graded according to the Common Terminology Criteria for Adverse Events of the National Cancer Institute (CTCAE) [18]. Post-remission therapy was in accordance with previously published data [19].

DNA extraction from AML cells was performed using the QIAamp DNA blood mini kit (Qiagen, Hilden, Germany). Real-time PCR was performed with the Universal Master Mix and SNP assays supplied by Applied Biosystems (Applied Biosystems, Foster City, CA, USA). A total of 48 SNPs, located within the genes of 7 different ABC transporters (*ABCA2*, *ABCA3*, *ABCB1*, *ABCB2*, *ABCB5*, *ABCB7*, and *ABCC1*) were investigated (Supplementary Table 1). Each SNP was analyzed with a single-tube assay. The identification of the corresponding SNPs was performed as in silico analysis using NIH dbSNP database and HapMap, respectively. Genotyping of the observed SNP was performed using the 7500 Sequence Detection Software (Version 1.3.1) by Applied Biosystems (Applied Biosystems, Foster City, CA, USA). Hereby the detection of each SNP variant was based on the fluorescence of associated markers (VIC or FAM as reporter markers). Statistical analyses on the impact of different SNP genotypes in ABC transporter genes were performed using SPSS (Version 19.0, Chicago, IL, USA).

Continuous variables were analyzed using the *U* test according to Mann-Whitney while categorical variables were analyzed with the *χ*2 test, respectively. Calculation of survival probabilities was performed according to the method of Kaplan-Meier. Overall survival (OS) and disease-free survival (DFS) were defined according to standard criteria [17]. The differences in OS and DFS for the respective genotypes of the SNPs were analyzed with the log rank test. Cox regression was applied to identify independent prognostic variables for survival in univariate and multivariable analyses. The significance level was terminated at 0.05. The 95% confidence interval (CI) of hazard ratios (HR) was computed to provide quantitative information on the relevance of results. The deviation from the Hardy-Weinberg equilibrium was analyzed using the *χ*2 test. For pairwise linkage disequilibrium between the genetic markers, the three estimators D, D′, and *r* were calculated. These analyses were carried out using the free statistical computing environment R (Version 2.3.1) and its library genetics for genetic analyses.

## Results

A significant impact on major outcomes (OS and DFS) was detected for three *ABCC1* (*MRP1*) transporter SNPs rs129081 (CACCCC[C/G]ACTCCA), rs212090 (TTACTG[A/T]TCCCAC), and rs212091 (ACCTTA[A/G]AGAACA).

**Figure 1:**
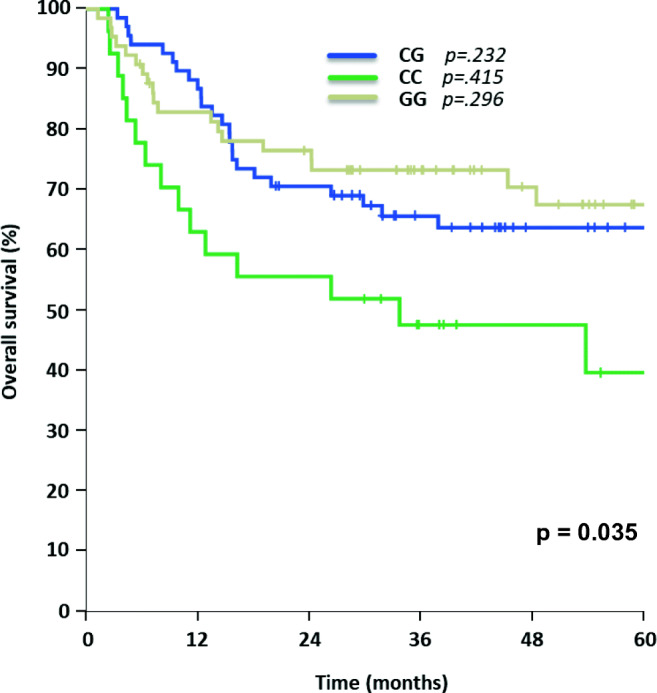
Overall survival for AML patients with different allele variants of rs129081

**Fig. 2:**
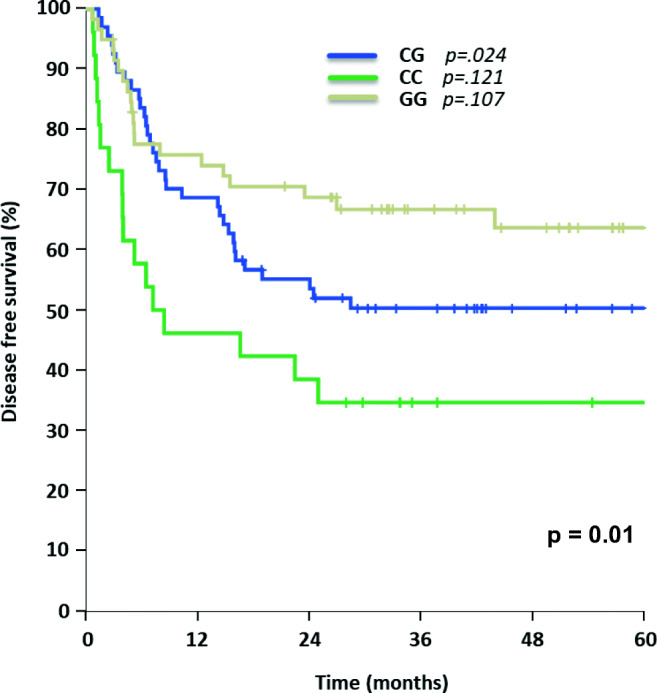
Disease-free survival for AML patients carrying allele variants of rs129081

Patients carrying the homozygous rs129081 GG-SNP (*n* = 65) had a significant higher 5-year-OS and 5-year-DFS, compared to the homozygous wild type CC (*n* = 27) and heterozygous CG (*n* = 68) patients (OS: GG 68% [95% CI 55–80%] vs. CC 40% [95% CI 18–61%] vs. CG 64% [95% CI 52–75%], *p* = 0.035; DFS: GG 64% [95% CI 50–77%] vs. CC 35% [95% CI 16–53%] vs. CG 50% [95% CI 38–62%], *p* = 0.01) in univariate analysis, Figs. 1 and 2. Although these results did not have a significant impact on OS in multivariable analysis (Table 2), heterozygous CG demonstrated a significant impact on DFS in multivariable analysis (*p* = 0.024), while the other allele variants of rs129081 had no independent influence on DFS (Table 3).

**Table 2:** Multivariate analysis—overall survival

SNP	Hazard ratio (HR)	95% CI	*p* value
rs129081			
CG	Baseline		0.232
CC	1.346	0.659–2.750	0.415
GG	0.716	0.383–1.340	0.296
rs212090			
AA	Baseline		0.590
AT	1.165	0.569–2.387	0.676
TT	1.469	0.678–3.180	0.329
rs212091			
GG	*Baseline*		0.044
AG	0.363	0.127–1.040	0.059
AA	0.296	0.113–0.774	0.013

Results of multivariate testing for overall survival of rs129081, rs212090, and rs212091 including hazard ratios, 95% CIs, and *p* values. CI, confidence interval

**Table 3:** Multivariate analysis—disease-free survival

SNP	Hazard ratio (HR)	95% CI	*p* value
rs129081			
CG	Baseline		0.024
CC	1.647	0.876–3.095	0.121
GG	0.626	0.354–1.107	0.107
rs212090			
AA	Baseline		0.089
AT	1.712	0.841–3.481	0.138
TT	2.321	1.092–4.932	0.029
rs212091			
GG	Baseline		0.058
AG	0.312	0.101–0.958	0.042
AA	0.280	0.098–0.797	0.017

Results of multivariate testing for disease free survival of rs129081, rs212090, and rs212091 including hazard ratios, 95% CIs, and *p* values. CI, confidence interval

**Fig. 3:**
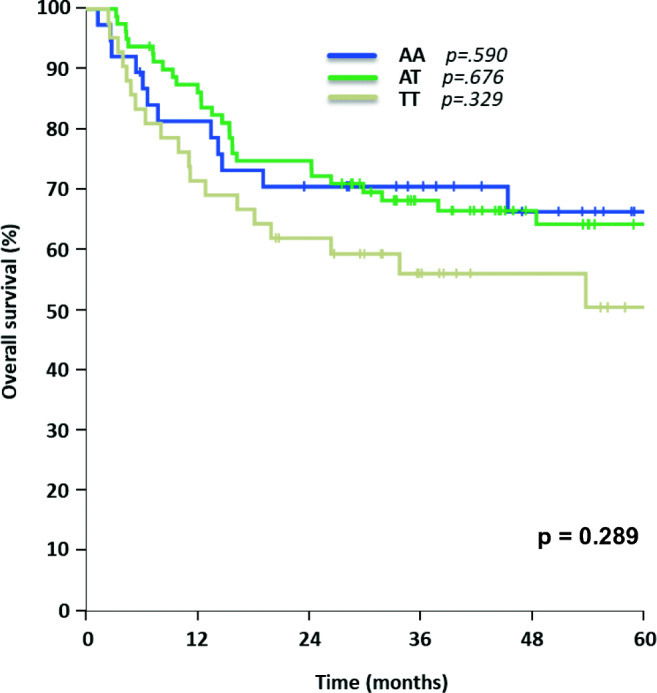
Overall survival for AML patients concerning different rs212090 allele variants

**Fig. 4:**
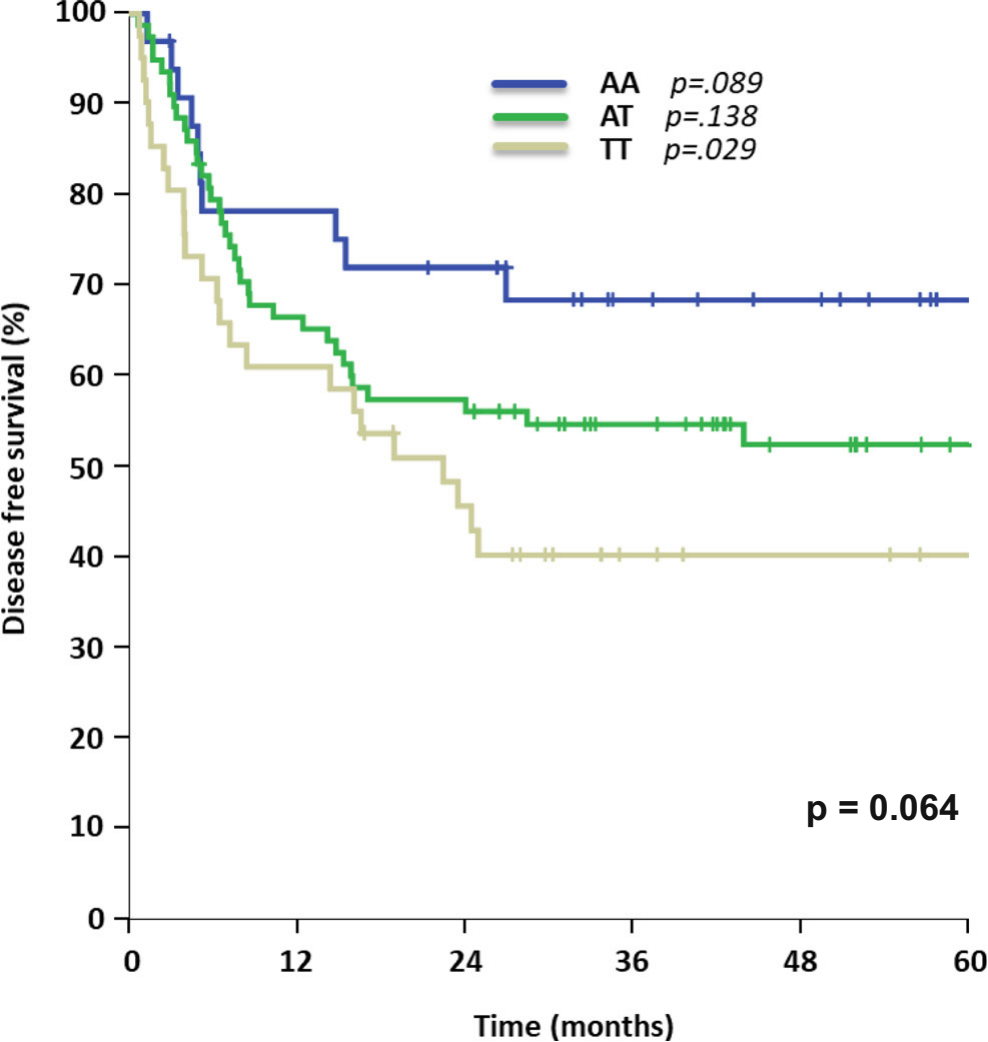
Disease-free survival for AML patients with different rs212090 allele expressions

For allele variants of SNP rs212090 (AA *n* = 38, AT *n* = 80, TT *n* = 42), no statistically significant impact on OS both in univariate and multivariate testing was observed (AA 66% [95% CI 50–82%] vs. AT 64% [95% CI 53–75%] vs. TT 50% [95% CI 33–68%], *p* = 0.289, Fig. 3 and Table 2), but a trend towards worse OS for genotype TT could be identified. Although rs212090 showed no significant influence on DFS with genotype TT leading to shortest DFS (AA 68% [95% CI 52–83%] vs. AT 52% [95% CI 41–64%] vs. TT 40% [95% CI 25–55%], *p* = 0.064, Fig. 4), it revealed a significant difference in DFS when comparing homozygous alleles AA and TT (*p* = 0.021) in univariate testing. The significant impact of homozygous variant TT of rs212090 on DFS was confirmed in multivariate analysis (*p* = 0.029, HR 2.321, 95% CI 1.092–4.932, Table 3), respectively.

**Fig. 5:**
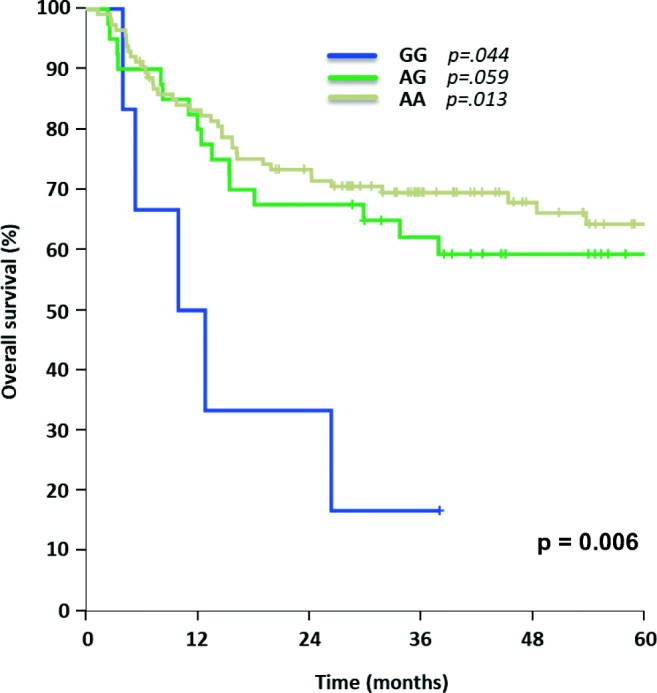
Overall survival for AML patients carrying different allele variants of rs212091

**Fig. 6:**
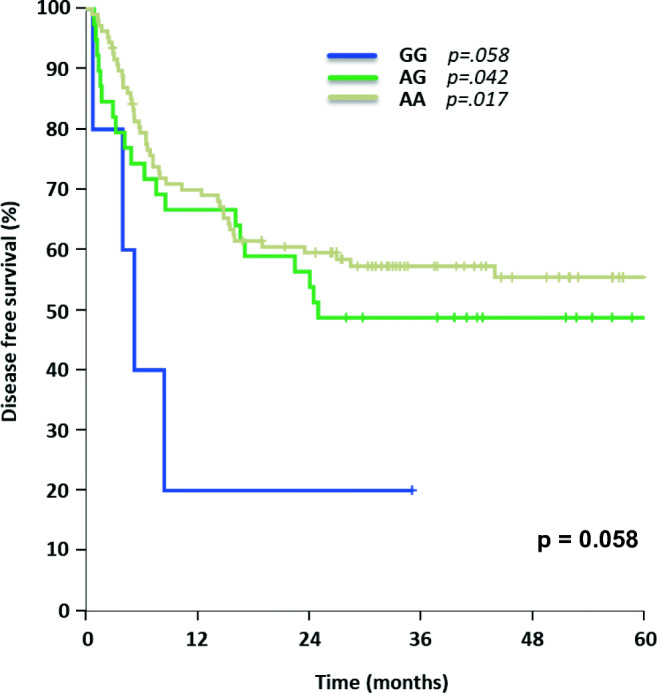
Disease-free survival for AML patients with different rs212091 allele expressions

SNP rs212091 showed a significant difference for OS with the homozygous allele GG (*n* = 6) leading to inferior OS (GG 0% vs. AA 64% [95% CI 55–74%] vs. AG 59% [95% CI 44–75%], *p* = 0.006, Fig. 5). In multivariable testing, an independent influence on OS for AA (*n* = 114, *p* = 0.044) and GG (HR = 0.296, 95% CI 0.113–0.774, *p* = 0.013) was demonstrated (Table 2). Regarding DFS for rs212091, no significant influence could be demonstrated in a univariate model (AA 55% [95% CI 46–65%] vs. 49% AG [95% CI 33–64%] vs. GG 0%, *p* = 0.058, Fig. 6). Comparing the homozygous genotypes of rs212091, GG was correlated with a significant reduction of DFS in contrast to homozygous counterpart AA (GG 0% vs. AA 55%, *p* = 0.018). These findings could be confirmed in multivariable analysis with independent significant impact on DFS for GG (HR = 0.28, 95% CI 0.098–0.797, *p* = 0.017) and AG (HR = 0.312, 95% CI 0.101–0.958, *p* = 0.042), Table 3.

A total of 78% (*n* = 125) of the here analyzed study cohort achieved CR. CR rate was not affected by these *MRP1* polymorphisms (rs129081 *p* = 0.078, rs212090 *p* = 0.148, rs212091 *p* = 0.420). A trend towards a higher incidence of relapse of the investigated SNP variants was in accordance with worse OS and DFS, respectively, but did not reach statistical significance (*p* = 0.152, *p* = 0.426, and *p* = 0.231).

Furthermore, our data also showed that there were no significant differences of standard clinical baseline characteristics, *FLT3*-ITD, or *NPM1* mutation status in the above described SNP groups. Regarding the combination of a co-occurring *FLT3*-ITD and *NPM1* mutation, rs129081, rs212090, and rs212091 allele variants were not significantly associated with a *FLT3*-ITD/*NPM1* co-expression (Table 4). Though SNP rs212090 alleles were characterized with significant differences in *FLT3*-ITD ratio, differences in *FLT3*-ITD ratios of rs129081 and rs212091 did not reach statistical significance (Table 4).

**Table 4:** AML risk factors

	rs129081				rs212090				rs212091			
	CC	CG	GG		AA	AT	TT		AA	AG	GG	
*FLT3-*ITD mut (%)	59.3	38.2	40	*p =* 0*.*15	39.5	41.3	47.6	*p =* 0.73	42.1	40	66.7	*p* = 0.46
*NPM1* mut (%)	44.4	55.9	60	*p =* 0*.*39	63.2	56.3	47.6	*p =* 0.37	59.6	45	50	*p* = 0.27
*FLT3-*ITD/*NPM1* (%)	33.3	27.9	27.7	*p* = 0.28	28.9	28.7	28.6	*p* = 0.77	27.2	30	50	*p* = 0.11
*FLT3-*ITD ratio > 0.5 (%)	88	54	65	*p =* 0*.*08	73	48	90	*p =* 0.01	69	50	100	*p =* 0.13
LDH (IU/l)	1126	788	688.5	*p =* 0.06	662.8	769.1	1001.2	*p =* 0.36	761.9	916.1	909.3	*p* = 0.97
WBC (×10^9^/l)	86.3	49.6	58.9	*p* = 0.39	57.1	53.5	73.4	*p* = 0.47	60.7	58.2	48.5	*p =* 0.97

Characteristic risk factors for worse (*FLT3*-ITD mutation status, *FLT3*-ITD ratio > 0.5, high LDH count at diagnosis, and high WBC at diagnosis) and good prognosis (*NPM1* mutation status) in AML, and their frequency among the analyzed allele variants of SNP rs129081, rs212090, and rs212091 are listed above. *FLT3*-ITD, FMS-like tyrosine kinase 3 internal tandem duplication; *NPM1*, Nucleophosmin 1; *LDH*, lactat dehydrogenase; *WBC*, white blood count

The patients’ age had no influence on the investigated *MRP1* polymorphisms (rs129081: *p* = 0.962, rs212090: *p* = 0.585, rs212091: *p* = 0.706). The quality and intensity of toxicities according to CTCAE after chemotherapy were not significantly different between allele variants. Genotype frequencies of rs129081, rs212090, and rs212091 were in accordance with the Hardy-Weinberg equation. Calculation of the linkage disequilibrium identified linked SNPs (rs129081/rs212090 *p =* 0, D′ = 1; rs129081/rs212091 *p =* 0, D′ = 1; and rs212090/rs212091 *p =* 1.62*^*–14, D′ = 1, respectively).

## Discussion

As AML presents clinically as a very heterogeneous hematopoietic malignancy with considerable variations in treatment response and patient outcome, prognostic markers need to be identified to offer individualized and risk-stratified therapy options to improve survival. Therapy resistance with progressive disease and limited therapeutic options still remains a major obstacle in the treatment of AML patients. Especially younger AML patients which represent a population with curative potential and long-term remission after intensive treatment, still need further adjusted treatment, as a considerable number of younger patients also suffer from progressive disease and relapse, but are uniformly treated with daunorubicin and cytarabine [20]. The clinical dilemma and pressure is about identifying mechanisms of these chemotherapy-resistances. During the last decade, several molecular prognostic factors for AML have been detected and included in standard routine diagnostics leading to improved risk stratification and prognosis evaluation and therefore adjusted therapeutic approaches [21–23]. With this analysis, we identified promising predictors for OS and DFS with SNP rs129081, rs212090, and rs212091 in NK-AML patients and indicate these SNPs to be prognostic markers for OS and DFS, respectively.

*MRP1* SNPs rs129081, rs212090, and rs212091 are located within the 3′UTR region of the *MRP1* transporter gene and therefore cause no primary change in amino acid sequence. As the 3′UTR region is a binding site for regulatory miRNAs, the influence of these SNPs by altering DNA sequence could unfold on a post-transcriptional level with different miRNAs leading to modification of the synthesized DNA strand and hereby potentially altering protein structures of ABC transporters after DNA transcription. In solid tumors as well as in AML, miRNAs have been identified as critical multiple key elements in tumorigenesis [24]. Whether these differences in survival are due to differential sensitivity towards anthracyclines, altered substrate-susceptibility or substrate binding-sites, or whether they can be applied to other functional and structural properties still remains unclear and needs further exploration. If chemotherapeutic agents themselves induce increased expression of transporter genes or increased transcription activity is also still a matter of investigation.

Interestingly, these polymorphisms did not confer any differences with regard to other AML specific characteristics and known risk factors for worse survival like secondary AML, white blood count, serum LDH, *FLT3*-ITD, or *NPM1*-mutation status at diagnosis. There were no differences concerning CR rates in these three *MRP1* SNP patient cohorts. Though CR was not significantly affected, genotypes CC for rs129081, TT for rs212090, and GG for rs212091 predicted inferior OS and DFS. These findings are in concordance with data by Schaich et al., who demonstrated an influence of *MRP1* gene expression on DFS, but not on CR [25]. Post-remission therapy had also no significant impact on outcome. Though a trend towards worse outcome and a higher incidence of relapse could be associated with *MRP1* polymorphisms, validation of our data in an independent AML cohort is necessary to confirm these observations for further research.

Moreover, no significant effect of these SNPs on chemotherapy-related toxicities was seen. In contrast, Cao et al. demonstrated an association of *MRP1* SNPs with gastrointestinal toxicities after chemotherapy in a Chinese population, which we could not confirm in our study of Caucasian patients [26]. No correlation was observed regarding age, gender, ECOG performance status, FAB subtypes, bone marrow blasts at diagnosis, or baseline blood count parameters (data not shown). As these three *MRP1* SNPs were in allele frequency accordance to healthy Caucasian individuals (data from dbSNP), we suggest a genetic predisposition for altered chemotherapy response, therapy resistance, and worse survival. The identification of linked polymorphisms indicates the influence of certain SNP haplotypes on therapy sensitivity and outcome in AML therapy.

On a molecular level, we hypothesize these SNPs as possible causal factors for differential anthracycline susceptibility. Therapy resistance conferred by *MRP1* SNPs might be an effect dominant in relapsed or progressive disease, when sensitive AML subclones are eliminated by cytotoxic chemotherapy and resistant blasts expressing *MRP1* SNPs proliferate in contrast to their chemosensitive counterparts. ABC transporter-associated MDR via drug efflux could be addressed in several studies [27, 28]. Therefore, AML patients carrying these genotypes associated with a poor prognosis may benefit from alternative therapeutic consolidation strategies like early allogeneic stem cell transplantation, higher dosages of daunorubicin, or alternative formulations of daunorubicin (e.g., CPX-351) during induction treatment [29]. Recently, higher dosages of daunorubicin during induction chemotherapy have also been demonstrated to be beneficial in certain subgroups of AML patients [30]. Therapeutic strategies trying to modulate the efflux of these transporters have failed in most clinical AML trials so far [31].

In conclusion, we demonstrate the influence of *MRP1* transporter polymorphisms CG of rs129081 on DFS, rs212090 TT allelic variant on DFS, and homozygous allele expressions GG and AA of rs212091 on OS, as well as AA and AG allelic rs212091 variants on DFS in AML patients with a normal karyotype for the first time to the best of our knowledge. Hence, we suggest these SNPs to be independent prognostic markers for AML concerning survival. As SNPs in miRNA binding sites may have an effect on gene transcription and protein expression, certain SNPs influencing prognosis in AML should be evaluated in addition to established mutational changes in AML to improve prognostic algorithms and individual treatment intensity and survival of AML patients.

## Electronic supplementary material

ESM 1 (DOCX 34 kb)
