# Supplementary material for: Multidrug-related protein 1 (MRP1) polymorphisms rs129081, rs212090, and rs212091 predict survival in normal karyotype acute myeloid leukemia
Source: Ann Hematol. 2020 Jul 3;99(9):2173–80. doi: 10.1007/s00277-020-04163-7 (PMC7419446; doi:10.1007/s00277-020-04163-7)
Supplement: Supplementary file 1 — (DOCX 34 kb) [file 277_2020_4163_MOESM1_ESM.docx]

**Supplement**

**Supplementary Table 1**

| **dbSNP** | **Assay ID** | **Transporter** |
| --- | --- | --- |
| rs13301354 | C__11569867_10 | ABCA2 |
| rs28381797 | AHI1OTT | ABCB1 |
| rs7036668 | AHHSQNL | ABCA2 |
| rs78787906 | AHGJSHD | ABCA2 |
| rs4335222 | C___495201_20 | ABCA2 |
| rs34039859 | C___243438_10 | ABCA2 |
| rs17793970 | C__34046152_10 | ABCA3 |
| rs45592239 | C__86551611_10 | ABCA3 |
| rs17064 | C__11711729_10 | ABCB1 |
| rs3842 | C__11711730_20 | ABCB1 |
| rs9282564 | C___2614970_10 | ABCB1 |
| rs2235036 | C__15951371_20 | ABCB1 |
| rs3216794 | C__34171753_10 | ABCB2 |
| rs241420 | C____549897_30 | ABCB2 |
| rs991760 | C___2961758_30 | ABCB2 |
| rs1057141 | C____549926_20 | ABCB2 |
| rs2127679 | C__15746839_20 | ABCB2 |
| rs41550019 | C__15746885_10 | ABCB2 |
| rs41561219 | C__25630686_20 | ABCB2 |
| rs1135216 | C__531909_20 | ABCB2 |
| rs1057373 | C___8848979_20 | ABCB2 |
| rs3198005 | C__27467792_10 | ABCB2 |
| rs62453384 | C__88810282_10 | ABCB5 |
| rs17143304 | C__25620999_10 | ABCB5 |
| rs6461515 | C__25621077_20 | ABCB5 |
| rs3210441 | C__27484550_10 | ABCB5 |
| rs966717 | C___7599509_10 | ABCB5 |
| rs117454564 | AHQJGPJ | ABCB5 |
| rs75464175 | AHRSEVR | ABCB5 |
| rs76879589 | AHS1C1Z | ABCB5 |
| rs76284767 | AHUAA77 | ABCB5 |
| rs74552040 | AHY93XO | ABCB5 |
| rs61732039 | AHVI9EF | ABCB5 |
| rs34603556 | C__25621025_20 | ABCB5 |
| rs2301641 | C___2544197_10 | ABCB5 |
| rs117497357 | AHX05QV | ABCB5 |
| rs1133577 | C___7422630_20 | ABCB7 |
| rs45492100 | AHY93W3 | ABCC1 |
| rs8187848 | C__30634107_10 | ABCC1 |
| rs45511401 | AH0I13B | ABCC1 |
| rs4148356 | C__25614385_20 | ABCC1 |
| rs74985930 | AH5IUSS | ABCC1 |
| rs28364006 | C__60277100_10 | ABCC1 |
| rs3743527 | C___8934057_30 | ABCC1 |
| rs129081 | AH20YFR | ABCC1 |
| rs212090 | C___3188829_30 | ABCC1 |
| rs4148380 | C__30634105_10 | ABCC1 |
| rs212091 | C___1003625_20 | ABCC1 |

**Supplementary Table 1.** List of SNP-assays supplied by Applied Biosystems including reference sequence (rs) by dbSNP, assay numbers and associated ABC-transporter gene.
